# dbEMT: an epithelial-mesenchymal transition associated gene resource

**DOI:** 10.1038/srep11459

**Published:** 2015-06-23

**Authors:** Min Zhao, Lei Kong, Yining Liu, Hong Qu

**Affiliations:** 1 School of Engineering, Faculty of Science, Health, Education and Engineering, University of the Sunshine Coast, Maroochydore DC, Queensland, 4558, Australia; 2 Center for Bioinformatics, State Key Laboratory of Protein and Plant Gene Research, College of Life Sciences, Peking University, Beijing 100871, P.R. China

## Abstract

As a cellular process that changes epithelial cells to mesenchymal cells, Epithelial-mesenchymal transition (EMT) plays important roles in development and cancer metastasis. Recent studies on cancer metastasis have identified many new susceptibility genes that control this transition. However, there is no comprehensive resource for EMT by integrating various genetic studies and the relationship between EMT and the risk of complex diseases such as cancer are still unclear. To investigate the cellular complexity of EMT, we have constructed dbEMT (http://dbemt.bioinfo-minzhao.org/), the first literature-based gene resource for exploring EMT-related human genes. We manually curated 377 experimentally verified genes from literature. Functional analyses highlighted the prominent role of proteoglycans in tumor metastatic cascades. In addition, the disease enrichment analysis provides a clue for the potential transformation in affected tissues or cells in Alzheimer’s disease and Type 2 Diabetes. Moreover, the global mutation pattern of EMT-related genes across multiple cancers may reveal common cancer metastasis mechanisms. Our further reconstruction of the EMT-related protein-protein interaction network uncovered a highly modular structure. These results illustrate the importance of dbEMT to our understanding of cell development and cancer metastasis, and also highlight the utility of dbEMT for elucidating the functions of EMT-related genes.

The epithelial-mesenchymal transition or transformation (EMT) refers to the change of epithelial cells to mesenchymal cells, which are critical for tissue development and initiation of metastasis for cancer progression^1^,^2^,^3^. Epithelial and mesenchymal cells differ in phenotype as well as function. Epithelial cells often closely connect to one another by cellular junctions such as tight junctions. In addition, they often show apico-basal polarity and polarization of the actin cytoskeleton. In contrast, mesenchymal cells link to each other by focal points and lack cellular polarization. During the EMT process, epithelial cells usually lose their tight cell connection and cellular polarity. As a result, a small number of epithelial cells can become migratory and invasive properties to transform into mesenchymal cells. These morphological and cellular changes during EMT have profound impacts on development, fibrosis and wound healing, and cancer progression^2^,^4^.

The importance of EMT was first recognized in embryonic development^5^,^6^. It is well acknowledged that EMT is associated with the initiation of placenta formation in the early stage of embryogenesis. After EMT, trophoectoderm cells are able to invade the endometrium and appropriate placenta placement; this enhances the exchange of gas and nutrients to the embryo. In addition, EMT is also involved in gastrulation during the later stage of embryogenesis. In this process, the cells that undergo EMT are able to ingress in the primitive streak of amniotes. In higher chordates, the mesenchymal cells that changed from the epithelial cells of primitive streak can migrate to forge the somites and interact with neural crest mesenchymal cells to form the heart mesoderm^1^. In vertebrates, epithelial and mesenchymal cells are the basic tissue units. During embryogenesis, epithelial cells of neuroectoderm may change to mesenchymal neural crest cells. As a result, these migratory mesenchymes can dissociate from neural folds and differentiate to specific cell types after travelling to specific target regions in the embryo.

Recently, the relationship between EMT and cancer has become increasingly clear from numerous studies^7^. EMT can assist the invasion during initiation of cancer metastasis. Cancer cells in primary sites can lose cell connection which increases their invasive properties, and they can enter the bloodstream through intravasation. Subsequently, these circulating tumor cells (CTCs) can colonize at new metastatic sites by forming micro-metastases. In addition, there is increasing evidence that shows that cells undergoing EMT have stem cell-like properties^8^. These cellular processes conferred by EMT are harmful for the patient not only because they enable the cancer cells to circulate with bloodstream but also because they reflect the properties of stemness that promote proliferation of cancer cells^4^.

Although essential in our understanding of the development of complex diseases, the genetic and biological information is scattered in the literature. To the best of our knowledge, there has been no systematic review of this literature to help us understand the molecular mechanisms underlying initiation of metastasis as it relates to cancer progression and the associated development processes. A comprehensive gene resource is needed to generate a complete molecular picture for EMT and associated diseases. In this study, we present the database dbEMT, the first literature-based EMT-related gene resource that serves as a reference dataset for understanding the cellular mechanisms of EMT-related processes such as initiation of metastasis for cancer progression. The resultant gene list in dbEMT and containing additional functional and genetic information will be a valuable resource for the EMT research community. Moreover, our systematic pathway and disease enrichment analyses reveal that the EMT-related genes enriched in multiple signal events are involved with many cancers and developmental processes. We believe that, dbEMT is the first example of an integrated and comprehensive gene resource to help elucidate the relationship between EMT and development and cancers. This database could have profound implications for the diagnosis, treatment and prevention of EMT-related diseases such as cancer metastasis.

## Results

### Functional enrichment analysis revealed fundamental role of proteoglycans in EMT

Gene-set enrichment analyses have been widely used to help biologists in the analysis and interpretation of the results from any interesting gene list^9^. In this article, the implemented hypergeometric tests were adapted to characterize whether an input gene-set had a different frequency of annotation pairs unlikely by chance, given the background genes sizes. The expected frequency was further obtained for each of the functional terms. Using protein-coding genes in the human as background, we identified 62 statistically significant enriched pathways (Table 1) and 28 diseases (Table 2) associated with the 377 genes in dbEMT. The enriched functional pathways are mainly related to cancer pathways such as TGF-beta signalling, proteoglycans in cancer, colorectal cancer, and pancreatic cancer (Table 1). In addition to cancer-related pathways, the genes were also highly enriched in cell surface interaction such as non-integrin membrane-ECM interaction, extracellular matrix organization, focal adhesion, and adhesion junction. These results show that multiple membrane-related signaling events are related to EMT, especially proteoglycans in cancer. Specifically, there are 65 genes in our dbEMT that are reported to relate to proteoglycans in cancer with a corrected *P-*value of 5.95E-11. Proteoglycan content and distribution are markedly altered during cancer progression^10^. The specific structure of proteoglycan allows it to interact with both the ligands and receptors that regulate cancer pathogenesis. Therefore proteoglycan, as well as glycosaminoglycans, often have important function in the tumor metastatic cascade by modulating key downstream signaling mediators such as epidermal growth factor receptor, insulin growth factor receptor, estrogen receptors, and Wnt members^11^. In summary, the level of complexity of cell surface signaling systems involved in EMT arises from their fundamental roles in response to microenvironment regulation.

### Enrichment diseases on the 377 EMT-related genes

**Figure 1:**
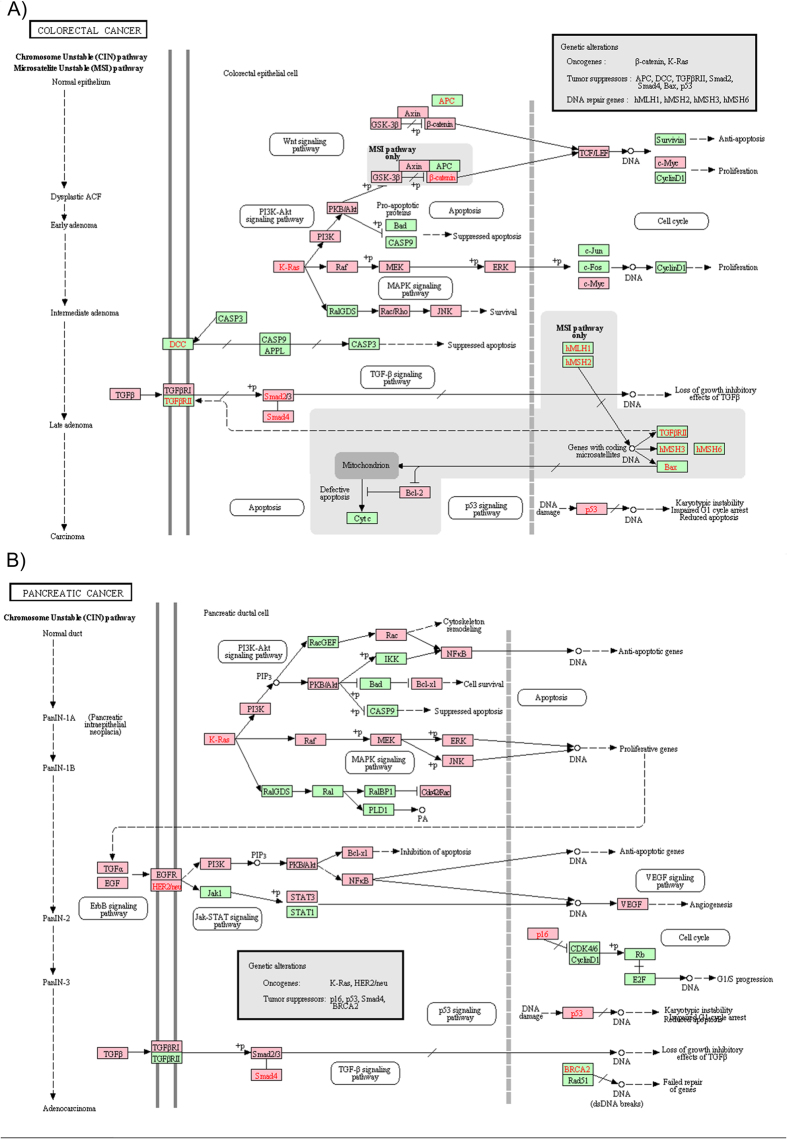
The highlighted mutated EMT-related genes in colorectal and pancreatic cancers. (**a**) The KEGG signaling map for colorectal cancer; (**b**) The KEGG signaling map for pancreatic cancer. The genes with pink color in two maps are detected in our dbEMT; the remaining green genes are human genes not in our dbEMT."

As a fundamental role on cell destination, it is not surprising that the EMT-related genes are consistently related to a number of complex diseases. However, the enrichment analysis of an EMT-related gene does not reveal if EMT has contributed to a specific disease. It does allow to test the risk factor of EMT in relevant diseases. The enrichment analysis on a few disease-related databases has confirmed that the 377 genes are related to a broad-spectrum of human cancers such as (Table 2). In total, 277 genes are related to various cancers. The cancers mainly occur in the digestive and urinary systems, including colorectal, pancreatic, endometrial, prostate, bladder, melanoma, renal cell, thyroid, oral, gastric, ovary, leukemia, cholangiocarcinoma, choriocarcinoma. Although many reports suggest that EMT is associated with cancer invasion and metastasis, there is no comparison of EMT across cancers. It is possible that the underlying common molecular mechanism between EMT and any cancers are the same. As shown in Fig. 1, there are 27 EMT-related genes in both colorectal cancer and pancreatic cancer. However, only 18 of these are common to both: SMAD2, SMAD3, SMAD4, PIK3CA, MAP2K1, TP53, AKT1, BRAF, TGFBR1, RAF1, TGFB3, TGFB2, MAPK3, MAPK1, MAPK8, KRAS, RAC1, TGFB1.

It is interesting to note that the 337 EMT-related genes are over-represented in the Alzheimer’s disease (AD) and type 2 diabetes (T2D). The possible association of EMT with Alzheimer’s disease is also supported by AD-specific biomarkers, which were selectively detected only in the affected AD brain^12^. The potential role of EMT in T2D was also found recently^13^. Although the direct association of AD and T2D with EMT has not reported. The study in adult human exocrine pancreatic cells revealed that the EMT could impact on dedifferentiation of ductal cells during the regeneration of type 2 diabetic pancreatic tissues. Therefore, further data mining on our dbEMT may provide a clue about a potential transformation in affected tissues or cells, including changes of upstream signaling and other cell surface interactions during the AD/T2D disease.

### Prioritizing the important genes in EMT and their mutational landscape in pan-cancer genomics data

The molecular basis that underlies EMT is unclear because of its high cellular complexity. Traditional characterization of candidate genes in individual studies often focuses on verifying specific genes functions related to EMT. However, the big map for the EMT and the relative importance for EMT-related genes have not been systematically evaluated. Using the gene ranking tool Endeavour, we obtained the top ten ranked genes involved inCTNNB1, SMAD3, HIF1A, TGFB1, ZEB2, EGFR, CDH1, ILK, ZEB1, TWIST1 (Table 3). Not surprisingly, majority of these top ranked genes are involved in the key pathway of EMT, such as the “Pathways in cancer”, “Adherens junction”, “Focal adhesion”, and “TGF-beta signaling pathway”.

**Figure 2:**
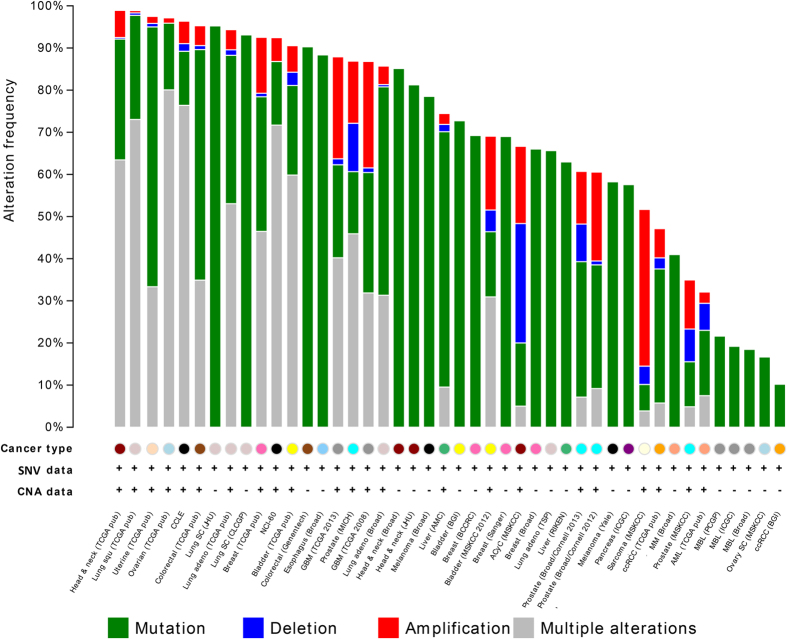
The mutational landscape for the top 100 EMT-related genes in multiple cancers.

Although EMT-related genes have been demonstrated to have abnormal gene expression or other functional relevance to cancer, the systematic examination of the genetic variants has not been conducted. These are useful for users to identify potential cancer metastasis genes for further screening. As shown in Fig. 2, the top 100 ranking EMT-related genes have tremendous mutations in cancers. It is interesting that the 100 genes are 98.9% mutated in both head/neck carcinomas (276 individuals) and lung squamous cell carcinomas (176 individuals). In 234 uterine cancer samples, there were more single nucleotide mutations for the patients. The most striking point is that 95.2% of the lung squamous cell carcinoma population from an analysis of 40 patients at the Johns Hopkins . with only single nucleotide mutations. The same observation can be found in colorectal cancer from the Genentech dataset (90.3% in 65 individuals) and esophagus cancer (88.4% in 129 individuals). In contrast, the three studies on medulloblastoma (MBL) only show less than 20% of mutation frequency in the designed case population. This suggests that MBL may have distinct metastasis mechanisms that are different from other cancers.

**Figure 3:**
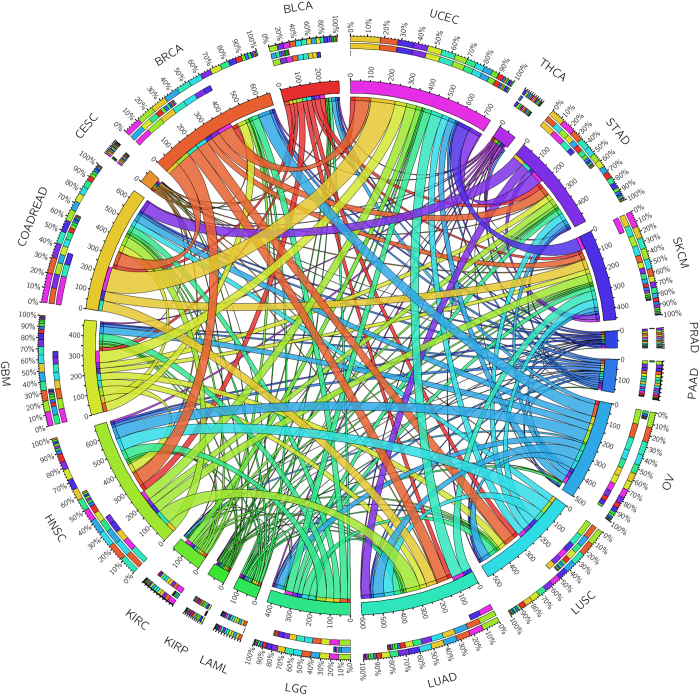
The shared somatic variants related to EMT across 19 cancer types. The length of circularly arranged segments is proportional to the total variants in each cancer type. The ribbons connecting different segments represent the number of shared variants between cancer types. The three outer rings are stacked bar plots that represent relative contribution of other cancer types to the cancer type’s totals.

To further explore the common and distinct EMT-related somatic variants in different cancer types, we mapped the 377 EMT-related genes to the mutation data of TCGA pan-cancer^14^. To focus on the functional variants, we excluded non-sense and silent mutations. The remaining contains mis-senses, splicings, frameshift SNVs and INDELs. As shown in the Fig. 3, majority of EMT-related somatic variants are shared between cancers. For instance, it is interesting that Uterine Corpus Endometrioid Carcinoma (UCEC) has many overlapping variants with colon and rectal adenocarcinoma (COADREAD) and suggests that the three cancers might have similar processes related to EMT. Or is simply because of the closeness of two organs. The similar comparison can be applied to other cancers. In summary, not all the cancers share the same set of EMT-related mutations. Considering both Figs 2 and 3, it is concluded that different cancers may have different mutation numbers and contents for EMT-related genes. According to the mutation frequency, some driver mutations occurred in some of critical genes, including MUC16 (1720 mutations), TP53 (1113 mutations), PTEN (1113 mutations), PIK3CA (291 mutations), NF1 (288 mutations), VCAN (274 mutations), ATM (236 mutations) and LAMA1 (230 mutations) in all the pan-cancer samples. These driver mutations in EMT-related pathway may induce the cancer cells in the progressive direction during EMT. To further test whether these top 100 EMT related-genes are mutated more frequently than expected by chance, we conducted 1000 resamplings to choose 100 mutated genes from 19 pan-cancer data. To evaluate the statistical significance, we checked each randomly selected gene set whether the number of genes with meaning mutations was more than the actual number of mutated genes in our top 100 EMT related-genes. Using the number of randomly selected node sets with more mutated genes as input, we calculated the empirical *P-*values (See Methods section). As shown in Table S2, all the 19 empirical *P-*values are all less than 0.01, which means the top 100 EMT related genes are highly mutated in the 19 pan-cancer datasets.

### Highly expressed and mutated EMT genes during stage III to stage IV in ovarian cancer

**Figure 4:**
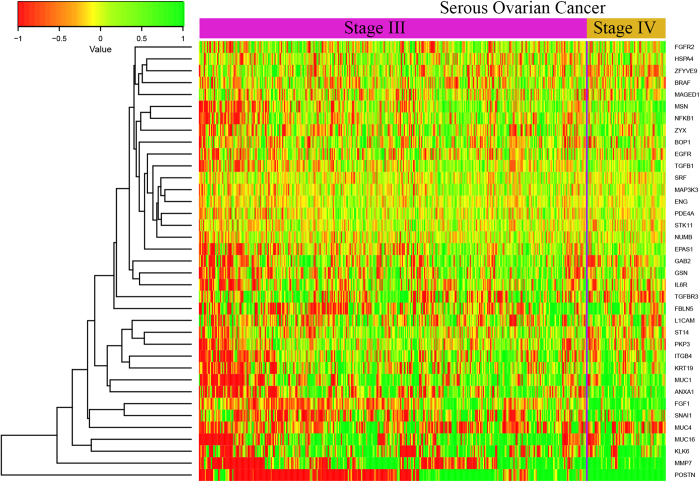
The heatmap for the 35 genes whose expression are increasing from stage III to stage IV in TCGA ovarian cancer samples.

As the EMT often occurs during the cancer metastasis, we only extracted those ovarian cancer samples related to stage III to stage IV, where metastasis transition occurs. The aim of this analysis was to identify those EMT-related genes whose expression is increasing between Stage III to stage IV. As shown in the Fig. 4, there are 35 mutated genes having comparatively high expression in average in ovarian cancer (student t test, *P-*values < 0.05). Multiple genes (MUC16^15^, POSTN^16^) have been reported to be related to ovarian cancer metastasis or cell movement. Our analysis reveals a similar relationship between periostin (POSTN) and EMT of ovarian cancer. Some of genes (e.g. MMP7) were reported to have potential biomarker role in ovarian cancer, but not for EMT process^17^. Those genes might be used as biomarkers for the ovarian cancer EMT-related process, especially, the strong different expression of periostin from stage III to IV which may indicate the ovarian cancer EMT process.

### The reconstructed EMT-related protein-protein interaction network exhibits a highly modular structure

**Figure 5:**
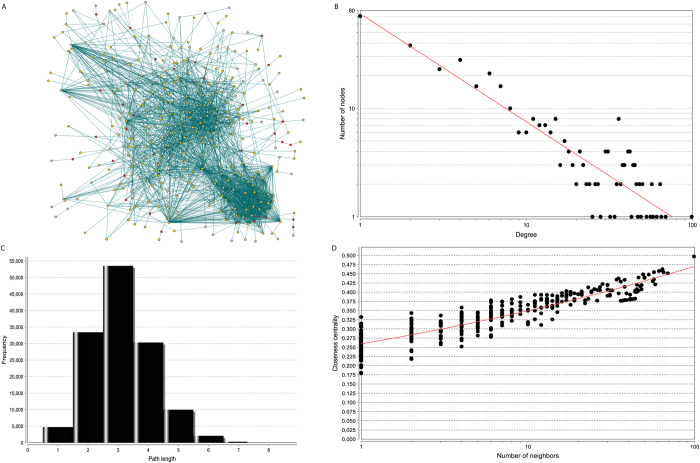
Reconstructed EMT map using protein-protein interaction data. (**A**) The 335 genes in orange are genes from the core dataset in our dbEMT. The remaining 36 genes in red are linker genes that bridge the 335 genes; (**B**) the degree distribution; (**C**) the short path length frequency; (**D**) the correlation between closeness centrality and the number of neighbors.

In the past decade, the knowledge of the cellular pathway for metabolism and signaling transduction have been summarized in a few popular biological pathway data such as the KEGG pathway. Recently, the Pathway Commons database combined all the popular pathway databases to provide gene-gene functional interaction pairs and these are useful for further pathway reconstruction^18^. In our study, we utilized reliable public data sources and constructed a more comprehensive cellular map for EMT. The reconstructed EMT contains 371 genes and 2357 gene-gene interactions is based on current evidence from known biological pathways (Fig. 5A). Of the 371 nodes, 335 of them are from our curated 377 IQ-related genes. The remaining 36 are the novel genes that may potentially bridge the EMT-related gene to fully implement their cellular function. The majority of curated EMT-related genes are linked to each other in a highly modular structure. This not only support the accuracy of our data, but it also reveals the EMT-related genes are highly connected to each other and form a high density cellular modular.

Further network topological analysis also indicates that most molecules in our map were closely connected. Only 80 nodes were without in-degree and out-degree. This means the majority of nodes can communicate with each other in short steps. The degrees of all nodes in our reconstructed EMT map follow a power law distribution *P(k) ~ k*^*-b*^, where *P(k)* is the probability that a molecule has connections with other *k* molecules and *b* is an exponent with an estimated value of 0.998. This means that our EMT map is different from all the human PPI network where most of nodes were sparsely connected with exponent *b* as 2.9^19^. This feature made the shortest path distribution for the entire network was a smaller number (2 to 4), and means that about 53% of the node communication can be reached only by three steps (Fig. 5C). This observation is also confirmed from the relation between closeness and the number of neighbors. As shown in Fig. 5D, the nodes with higher closeness are more likely to have more neighbors and vice versa. With high modularity, the hub nodes in this network may have prominent roles as common connections to mediate information transduction in the short path. In total, there are 13 genes with at least 50 connections and these are: SRC (99), EGFR (69), PTK2 (65), PRKCA (64), MAPK1 (64), AKT1 (61), JAK2 (58), CTNNB1 (57), ERBB2 (57), FGFR1 (56), MAPK3 (53), TP53 (50), MAPK14 (50). All 13 genes are from our literature-based gene set; none are linker genes. In summary, our reconstructed map not only discovers multiple paths related to a few known signaling pathways but also provides a broader picture for the highly modular structure of the different previously unconnected signaling pathways.

## Conclusion

dbEMT is constructed as a free database and analysis server to enable users to rapidly search and retrieve summarized EMT-related genes. The comprehensive functional enrichment analyses reveal that EMT-related genes are enriched in multiple signal events associated with development and cancers. Important questions should now be directed towards integration of various signaling pathways to initial EMT events. The dbEMT is freely available at http://dbemt.bioinfo-minzhao.org/.

## Methods

### Extensive literature search for EMT-related genes and gene curation

**Figure 6:**
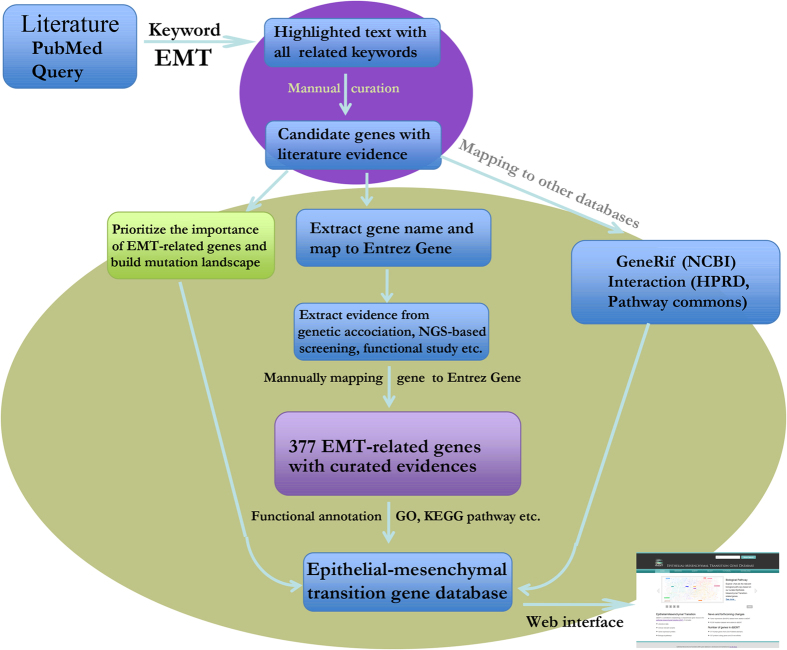
Pipeline for collection, expansion and annotation of EMT-related genes.

To obtain a precise EMT-related gene list with experimental evidence, we performed our literature search and curation as the following four steps (Fig. 6). Firstly, we first performed an extensive literature query against PubMed (on Dec 19^th^, 2013) using complex expression: ("Epithelial-mesenchymal transition"[Title/Abstract] OR "Epithelial mesenchymal transition"[Title/Abstract] OR "EMT"[Title/Abstract]) AND (("genome-wide association study" [Title/Abstract] OR "genome wide association study" [Title/Abstract]) OR ("gene"[Title/Abstract] AND ("association"[Title/Abstract] OR "microarray" [Title/Abstract] OR "expression" [Title/Abstract] OR "linkage" [Title/Abstract] OR "proteomics" [Title/Abstract] OR "genetic" [Title/Abstract] OR "metabolomics" [Title/Abstract] OR "copy number variation" [Title/Abstract] OR "idiopathic" [Title/Abstract] OR "hereditable" [Title/Abstract] OR "family" [Title/Abstract] OR "mouse model" [Title/Abstract] OR "animal model" [Title/Abstract] OR "microRNA" [Title/Abstract] OR "mutation" [Title/Abstract] OR "SNP" [Title/Abstract] OR "drug" [Title/Abstract] OR "transporter" [Title/Abstract]))). Secondly, 1507 abstracts were retrieved and grouped by the “Related Articles” function in Entrez system. Thirdly, we then extracted the EMT-related description from the grouped abstracts. Those sentences related to EMT were manually read to extract the gene names and organism information with experimental evidence. Lastly, the extracted candidate gene name and organism information were manually checked. Finally, the 377 Entrez human Gene IDs with high confidence were collected as core EMT-related genes. This core gene list will be regularly updated based on newly published literature.

### Biological functional annotations and database construction

To present the biological function involved and over-represented in our 377 EMT-related genes, we retrieved comprehensive functional information from public resources. The basic gene information and sequences are included and crosslinked to the NCBI Entrez gene^20^, UniProt^21^, Ensembl^22^ and Gene Ontology^23^. The mRNA expression profiling data from both normal and tumor tissues are imported from BioGPS^24^. To obtain comprehensive pathway-related information, we annotated the EMT-related genes using transporter substrate database^25^, BioCyc^26^, KEGG Pathway^27^, rate-limiting enzyme database^28^ PANTHER^29^, PID Curated^30^, pathway localization database^31^, PID Reactome^32^,^33^. The involved diseases were incorporated from GAD (gene association database)^34^, KEGG Disease^35^, Fundo^36^,^37^, NHGIR^38^, as well as OMIM^20^. In addition, the original EMT-related literature references in the NCBI PubMed database are hyperlinked to each gene. A semi-automatic annotation pipeline is implemented to integrate functional information from Gene annotation^39^, Gene Ontology annotation, HPRD/BIND/BioGRID interaction annotation, KEGG LIGAND/BioCarta signaling event annotation^40^,^41^, and OMIM annotation using Perl Script and Swiss knife module^42^,^43^,^44^,^45^.

To obtain updated relevant publications in future, we constructed an automatic literature searching terms using My NCBI tool, which will return matched articles every two weeks. We will consider using the Entrez literature similarity to cluster the newly available articles to facilitate the curation. In addition, to keep pace with fast generation of cancer genomic data, we have implemented an automatic system to import functional information from a variety of public data sources, which can help us integrate more annotations quickly. Once the data content is updated, the web interface will be updated accordingly annually.

### Gene set enrichment analysis

Throughout the paper, the functional enrichment analysis from KEGG, Reactome and other disease databases for each gene set were identified by KOBAS^46^. In these enrichment analyses, all the human protein-coding genes were set as background to calculate statistical significance. In addition, the Benjamini-Hochberg multiple testing corrected *P-*values for enriched annotations were adopted based on hypergeometric test by using KOBAS. Finally, the enriched human pathways with corrected *P-*values less than 0.01 were identified as over representative annotations for each gene set.

### Gene ranking using Endeavour and cancer mutation landscape

We performed a gene prioritization using the Endeavour web server^47^ to help the user prioritize all 377 genes in dbEMT. Endeavour utilizes multiple dimensional data to rank the input genes, including gene expression, regulatory information, functional annotations, sequence features, and literature mining data. It begins by extracting features about the genes that are known to play a role in the EMT. In this study, we compiled a training gene list that included 12 genes (TGFB1, SNAI1, CDH1, ZEB1, SNAI2, TWIST1, SMAD3, ILK, HIF1A, EGFR, ZEB2, and CTNNB1) with at least eight PubMed abstracts to train the ranking model. In the second stage, the ranking model was used to prioritize the remaining 365 genes using multiple genomic data. Finally, Endeavour combined all the rankings to a global ranking for all the candidate EMT-related genes using order statistics. In total, 209 valid human genes were ranked (Table S1). The top 100 ranking of EMT related-genes are input into the cBio portal to obtain a mutation pattern across multiple cancers^48^.

### Empirical resampling for evaluating the single nucleotide mutations of EMT related-genes across multiple cancers

To determine whether the SNV somatic mutation rate of top 100 EMT related-genes are relatively higher than expected, we conducted a total of 1000 resamplings. To this aim, we first downloaded all the SNV somatic mutations in MAF format across 19 TCGA tumor-types (https://www.synapse.org/#!Synapse:syn1729383)^49^. During each resampling process, 100 human genes were randomly selected from 20059 human genes with SNV somatic mutations. To provide insight into biological meaning, we only focused on somatic mutations containing mis-senses, splicings, frameshift SNVs and INDELs instead of non-sense and silent mutations. We repeated this random process 1000 times.

Next, we counted the number of randomly selected node sets (N) whose number of mutated genes was more than the actual number of mutated genes in the top 100 EMT related-genes. Finally, we calculated the empirical *P-*value using N/1000 to represent the significance of how the top 100 EMT related-genes are highly mutated in certain cancers. Based on this empirical resampling approach, the empirical *P-*values for the relationship between the top 100 EMT related-gene set and cancers are all less than 0.01 (Table S2).

### Gene expression analysis in ovarian cancer

To explore the gene expression changes during cancer metastasis, we focused on a gene expression profile with 489 high-grade serous OVC samples produced by using three gene expression microarray platforms (Affymetrix Exon 1.0 array, Agilent 244 K whole genome expression array, and Affymetrix HT-HG-U133A array)^50^. To present a unified gene expression, all the three datasets were normalized and the expression values were calculated for each sample and gene on each platform separately. After subtracting the mean value across samples for the same gene, the expression values were divided by the standard deviation across samples to obtain the relative gene expression scores. Finally, the relative expression data from three platforms was integrated into a unified data set of 11,864 genes using a factor analysis model without batch effects^51^,^52^,^53^. The processed final gene expression data was downloaded from the TCGA website in a format of matrix, in which one row for each gene and one column for each of the sample (https://tcga-data.nci.nih.gov/docs/publications/ov_2011/).

From the prepared gene expression matrix of ovarian cancer, we extracted the expression values of the mutated EMT related-genes in stage III and IV. In total, there are 65 EMT related-genes with somatic mutations in ovarian cancer. Next, we extracted all the 65 gene expression profiles from 381 stage III samples and 79 stage IV samples of ovarian cancer. We determined the gene expression changes of the transition between stage III and IV using the student test. Finally, we found 35 genes with paired t-test P-values less than 0.05. All the statistic tests and heatmap generations were using R programing environment (R Development Core Team 2008).

### Reconstructing a protein-protein interaction network related to EMT genes

To present the underlying biological mechanisms related to EMT genes, we extracted a protein-protein interacting relation between 377 EMT-related genes with the remaining human genes. To this end, we collected a non-redundant human interactome based on all the known protein-protein interactions using PathCommons, containing 3629 nodes and 36034 protein-protein links. It is noteworthy that the collected protein-protein interactions are from pathway databases (HumanCyc, NCI signaling pathway database, Reactome, and KEGG), which have biological significance. The final interactome represents pathway-based gene–gene interaction links. To extract a sub-network related to the 377 EMT-related genes of interest, we used the similar approach implemented in our previous study^54^. In this algorithm, all input genes were mapped to the human interactome, which was used to produce a sub-network with input genes connected by their shortest path.

In spite of its high complexity, the biological network often follows a few simple rules that may relate to its function^55^. Generally, the topological characters may give more clues to reveal the potential function for a network. To decompose the reconstructed interactome from our 377 EMT-related genes, topological analyses were conducted using the NetworkAnalyzer plugin in Cytoscape 2.8 (Fig. 5B–D)^56^. Often the amount of connections at each node was represented as degree in a network^55^. Finally, closeness centrality was used to reveal the shortest steps for one node to reach another^55^. The final network visualization was performed using Cytoscape^56^.

### Web interface development

**Figure 7:**
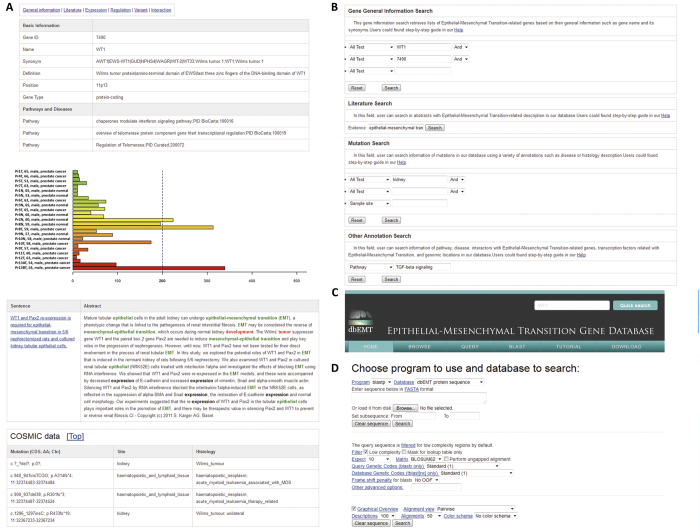
Web interface of dbEMT. (**A**) The basic information in each EMT-related gene page; (**B**) Query interface for text search; (**C**) BLAST search interface for comparing query against all sequences in dbEMT; (**D**) Browser interface for genes in top 10 enriched pathways, top 10 enriched diseases and shared cytobands.

The reliable open source relational database MySQL was used to store all the data and annotations in dbEMT on a Linux server. The CGI Web-based interface to the database is implemented using Perl. Using the Perl CGI module and JavaScript technology, web pages for each gene in the database are generated. In dbEMT, all the genes are annotated comprehensively with hyperlinks to their original data resources (Fig. 7A). Gene expression in various normal tissues and cancer samples are represented in a colored bar chart (Fig. 5A). In addition, the extensive literature evidence associated with EMT genes are also complied and highlighted with keywords related to EMT or diseases.

Our dbEMT provides a user-friendly web interface to perform text query (Fig. 7B), or to run a sequence similarity search (BLAST) against the nucleotide and protein sequences in dbEMT (Fig. 7C). In the text-based query page, six different powerful input forms are provided for the Entrez Gene ID, pathway and disease information, genomic location, literature evidence, and gene expression range in normal/cancer samples. Additionally, a quick text search for GeneID, gene symbol, and gene alias is at the top right of each page, and is convenient for a user to obtain any data in the database, especially literature-based annotations. Furthermore, users can browse the data in dbEMT in a variety of ways, including significantly enriched pathway, related disease, reported linkage region, and chromosome number (Fig. 7D). For each related KEGG pathway, the marked chart is provided to highlight the entire related EMT-related gene. Finally, for any advanced study, dbEMT provides all downloadable gene annotation and sequence information in a plain text format for all the collected 377 genes related to EMT.

## Additional Information

**How to cite this article**: Zhao, M. *et al.* dbEMT: an epithelial-mesenchymal transition associated gene resource. *Sci. Rep.* **5**, 11459; doi: 10.1038/srep11459 (2015).

## Supplementary Material

Supplementary Information

Supplementary Information

## Figures and Tables

**Table 1:** The statistically significant enriched pathways of EMT-related genes in core dataset from different pathway databases.

Pathway	Adjusted P-values*
**KEGG pathway**	
Proteoglycans in cancer	5.95E-11
Pathways in cancer	1.21E-09
Colorectal cancer	1.95E-07
Pancreatic cancer	5.05E-07
MicroRNAs in cancer	5.81E-07
Endometrial cancer	1.84E-06
Prostate cancer	5.90E-06
Chronic myeloid leukemia	1.66E-05
Bladder cancer	1.78E-05
TGF-beta signaling pathway	2.13E-05
Focal adhesion	4.17E-05
Adherens junction	4.36E-05
Hippo signaling pathway	6.08E-05
ErbB signaling pathway	0.000171393
Melanoma	0.000188448
Renal cell carcinoma	0.000211855
Thyroid cancer	0.000558926
Acute myeloid leukemia	0.000746089
Glioma	0.001053132
Prolactin signaling pathway	0.002672626
Non-small cell lung cancer	0.002846076
Hepatitis B	0.004685088
HIF-1 signaling pathway	0.005619905
VEGF signaling pathway	0.0089538
**Reactome pathway**	
Non-integrin membrane-ECM interactions	5.21E-06
Extracellular matrix organization	9.03E-06
Signaling by SCF-KIT	1.30E-05
Signaling by ERBB4	6.35E-05
Syndecan interactions	0.000155583
Signaling by FGFR in disease	0.000170943
GAB1 signalosome	0.000186436
Fc epsilon receptor (FCERI) signaling	0.000491729
Signaling by FGFR	0.000517638
Downstream signal transduction	0.000517638

^*^Adjusted *P*-values: the *P*-values of the hypergeometric test were corrected by Benjamini-Hochberg multiple testing correction.

**Table 2:** Significant enriched pathways in the reconstructed EMT-specific network.

Pathway	Adjusted P-values*
**KEGG disease**	
Cancers	2.29E-07
Cancers of the digestive system	3.52E-07
Cancers of the urinary system and male genital organs	6.52E-06
Gastric cancer	2.64E-05
Penile cancer	0.000250093
Cancers of the breast and female genital organs	0.000314144
Hepatocellular carcinoma	0.000375659
Cholangiocarcinoma	0.002488127
Head and neck cancers	0.003815715
Pancreatic cancer	0.003867987
Skin cancers	0.005592227
Choriocarcinoma	0.00804457
**GAD database**	
Prostate cancer	9.82E-06
Breast cancer	3.02E-05
Colorectal cancer	3.43E-05
Lung cancer	0.000106932
Stomach cancer	0.000271348
Bladder cancer	0.001245754
Kidney cancer	0.00169854
Ovarian cancer	0.003580256
Alzheimer's disease	0.004749989
Melanoma	0.004782684
Diabetes, type 2	0.005049578
Colorectal neoplasms	0.008608511
Endometrial cancer	0.009991705
Overall effect	0.009991705
**FunDO database**	
Oral cancer	0.002189338
Endometriosis	0.003400183

^*^Adjusted *P*-values: the *P*-values of the hypergeometric test were corrected by Benjamini-Hochberg multiple testing correction.

**Table 3:** The top 20 ranked EMT-related genes.

GeneSymbol (Ranked)	Global Rank P-value^1^	Global Rank ratio^2^
CTNNB1	5.62E-10	0.00291
SMAD3	7.17E-10	0.00581
HIF1A	3.34E-09	0.00872
TGFB1	8.90E-09	0.0116
ZEB2	1.06E-08	0.0145
EGFR	2.41E-08	0.0174
CDH1	4.24E-08	0.0203
ILK	5.13E-07	0.0233
ZEB1	1.04E-06	0.0262
TWIST1	7.58E-06	0.0291
SNAI2	8.77E-06	0.032
EPAS1	2.11E-05	0.0349
SMAD2	2.59E-05	0.0378
SNAI1	0.000157	0.0407
SMAD4	0.000247	0.0436
IGF1R	0.000723	0.0465
ERBB2	0.000819	0.0494
MET	0.00133	0.0523
AKT1	0.00191	0.0552
SMAD7	0.00313	0.0581

^1^Represent the probability that a candidate gene would obtain these ranks by chance.

^2^The global ranking ratio from Endeavour.
